# Analysis of Complete Nucleotide Sequences of Angolan Hepatitis B Virus Isolates Reveals the Existence of a Separate Lineage within Genotype E

**DOI:** 10.1371/journal.pone.0092223

**Published:** 2014-03-14

**Authors:** Barbara V. Lago, Francisco C. Mello, Flavia S. Ribas, Fatima Valente, Caroline C. Soares, Christian Niel, Selma A. Gomes

**Affiliations:** 1 Laboratory of Molecular Virology, Oswaldo Cruz Institute, FIOCRUZ, Rio de Janeiro, Brazil; 2 Eduardo dos Santos Foundation, Ministry of Health, Luanda, Angola; CRCL-INSERM, France

## Abstract

Hepatitis B virus genotype E (HBV/E) is highly prevalent in Western Africa. In this work, 30 HBV/E isolates from HBsAg positive Angolans (staff and visitors of a private hospital in Luanda) were genetically characterized: 16 of them were completely sequenced and the pre-S/S sequences of the remaining 14 were determined. A high proportion (12/30, 40%) of subjects tested positive for both HBsAg and anti-HBs markers. Deduced amino acid sequences revealed the existence of specific substitutions and deletions in the B- and T-cell epitopes of the surface antigen (pre-S1- and pre-S2 regions) of the virus isolates derived from 8/12 individuals with concurrent HBsAg/anti-HBs. Phylogenetic analysis performed with 231 HBV/E full-length sequences, including 16 from this study, showed that all isolates from Angola, Namibia and the Democratic Republic of Congo (n = 28) clustered in a separate lineage, divergent from the HBV/E isolates from nine other African countries, namely Cameroon, Central African Republic, Côte d'Ivoire, Ghana, Guinea, Madagascar, Niger, Nigeria and Sudan, with a Bayesian posterior probability of 1. Five specific mutations, namely small S protein T57I, polymerase Q177H, G245W and M612L, and X protein V30L, were observed in 79-96% of the isolates of the separate lineage, compared to a frequency of 0–12% among the other HBV/E African isolates.

## Introduction

Hepatitis B virus (HBV) infection still remains an important cause of morbidity and mortality worldwide, particularly in the Asian and African developing countries. Worldwide, about 350 million people are chronically infected and about 600,000 people die every year due to the consequences of acute or chronic HBV infection [1].

HBV has a DNA genome of about 3.2 kb, which contains four open reading frames, namely S (surface antigen), P (polymerase), C (core protein) and X (regulatory protein). Generally, the development of antibodies against hepatitis B surface antigen (HBsAg) leads to the viral clearance. However, the concurrent presence of HBsAg and anti-HBs has been reported occasionally [2]–[5]. Studies performed with HBV carriers testing positive for both serological markers have demonstrated that mutations in the pre-S [6], [7] and S [8], [9] regions of the genome may lead to changes in the immunogenicity of the viral particles, thus influencing the viral behavior and clinical course of the liver disease.

Africa is globally classified as a high HBV prevalence area, although hepatitis B endemicity may vary greatly from a region to another [10]. Based on HBsAg positivity, HBV is hyperendemic (>8%) in some sub-Saharan countries such as Nigeria, Namibia, Gabon and Cameroon. Other countries (Kenya, Zambia, Côte d'Ivoire, Liberia, Sierra Leone and Senegal) are considered areas of intermediate endemicity (2–8%), while Egypt, Tunisia, Algeria and Morocco, located at the North of the continent, are low endemicity (<2%) regions. Prevalence of hepatitis B core antibody (anti-HBc), a serological marker for previous HBV exposure, is extremely high (>80%) in various African populations [10].

Human HBV isolates have been classified into at least eight genotypes, denoted A (HBV/A) to H (HBV/H), based on a sequence divergence >7.5% in the entire genome [11]–[14]. Genotypes A–D and F have been divided into subgenotypes based on an intergroup divergence of about 4% [15]. Genotypes A, D and E are the most frequently found in Africa and show a characteristic distribution, with subgenotype A1 being prevalent in Southern and Eastern coastal regions, HBV/E spread in West Africa and genotype D prevailing at the North of the continent [10], [16].

The evolutionary history of HBV/E is still unclear. This genotype is largely spread in West Africa but shows a restricted genetic variability. Indeed, the mean divergence over the HBV/E whole genome does not exceed 1.75%, in comparison to 4% among HBV/A African isolates [17]. Despite the slave trade that lasted from the 16th to the 19th century, HBV/E has not been reported in the New World, except in people who maintained relations with Africa [18]. These findings support the hypothesis of a recent (<200 years) origin of HBV/E [17], [19].

A previous study has shown that genotype E is predominant in Angola [20]. In this study, we investigated the genetic diversity of Angolan HBV/E isolates and demonstrate the existence of a separate cluster within genotype E formed by isolates from Angola, Namibia and the Democratic Republic of Congo.

## Results

### Mutations in the Surface Antigen, Polymerase and Precore-Core Genes

No nucleotide sequence could be determined for five of the 35 Angolan virus isolates previously characterized as HBV/E, due to insufficient amounts of PCR products. The other 30 were genetically characterized. Full-length genome and pre-S/S sequences were determined for 16 and 14 isolates, respectively. Table 1 shows demographic, serological and occupational data of the 30 HBV/E infected individuals, as well as their hepatitis B risk factors (when known) and anti-HIV status. Only three persons (LDA265, LDA423 and LDA470) were aware of their HBV carrier status. Twelve (40%) subjects had concurrent HBsAg and anti-HBs, while the remaining 18 (60%) tested HBsAg positive and anti-HBs negative. All but one HBV/E strain belonged to serotype *ayw4*, on the basis of the deduced amino acid composition. The exception was the isolate LDA332 which showed a proline residue at position 127 in the small S protein, compatible with serotypes *ayw1* and *ayw2*. Twenty-nine out of 30 isolates showed a start codon Met83 in the pre-S1 region (not shown), which is a characteristics of genotype E and may result in the translation of an elongated middle S protein.

**Table 1:** Demographic, serological and occupational data and hepatitis B risk factors of the Angolan HBV/E carriers of this study.

Subject	Sex	Age (y)	Province of birth	Anti-HBs	Anti-HIV	Hospital position	Known hepatitis B risk factors
LDA067	F	29	Huambo	Pos	Neg	Yes (lab technician)	Surgery
LDA072	F	22	Luanda	Pos	Neg	No	–
LDA111	F	28	Cuanza Norte	Pos	Pos (untreated)	No	Blood transfusion
LDA154	M	20	Luanda	Neg	Neg	No	Tattoo
LDA173	M	37	Luanda	Neg	Pos (HAART)	No	–
LDA232	M	38	Cuanza Sul	Pos	Pos (untreated)	No	–
LDA264	M	34	Uíge	Neg	Neg	No	–
LDA265	M	44	Luanda	Pos	Pos (HAART)	No	Surgery
LDA274	F	36	Huila	Neg	Neg	Yes (nurse)	–
LDA277	F	68	Uíge	Pos	Neg	No	Blood transfusion
LDA278	M	26	Uíge	Pos	Neg	No	–
LDA332	M	43	Malange	Neg	Neg	No	–
LDA335	F	37	Luanda	Neg	Neg	No	–
LDA339	F	37	Luanda	Neg	Pos (HAART)	No	Surgery
LDA349	M	21	Luanda	Neg	Neg	No	–
LDA379	M	26	Luanda Norte	Neg	Neg	No	–
LDA386	M	20	Luanda	Neg	Neg	No	Multiple^a^
LDA399	F	23	Luanda	Neg	Neg	Yes (cleaner)	Tattoo
LDA405	M	21	Luanda	Neg	Neg	No	–
LDA408	F	21	Luanda	Neg	Neg	No	–
LDA417	F	26	Luanda	Neg	Neg	Yes (nurse)	–
LDA423	F	43	Luanda	Pos	Neg	No	–
LDA439	M	34	Huambo	Neg	ND	No	–
LDA445	F	23	Benguela	Neg	ND	No	–
LDA452	M	36	Bengo	Neg	ND	No	–
LDA470	M	28	Malanje	Pos	Neg	Yes (gardener)	Surgery
LDA481	M	43	Uíge	Pos	ND	Yes (porter)	Tattoo
LDA489	M	53	Bengo	Pos	Neg	Yes (nurse)	Multiple^b^
LDA494	M	39	Luanda	Pos	Neg	No	Tattoo
LDA504	F	39	Malanje	Neg	Neg	Yes (administrative)	–

a Multiple sexual partners, surgery, tattoo; ^b^ Surgery, blood transfusion; ND, not determined.

Deletions varying from one to 18 amino acids in length were found (Table 2). Among the 12 isolates derived from individuals with concurrent HBsAg/anti-HBs, five (42%) showed a deletion in their genome (one in the pre-S1- and four in the pre-S2 region). Only one deletion (amino acids residues 3–12 in the pre-S1 region) was observed among the 18 HBV isolates derived from anti-HBs negative subjects. Based on previous mapping of immune epitopes and functional domains within the pre-S region [21], [22], all five deletions present in isolates derived from individuals with concurrent HBsAg/anti-HBs disrupted T- and/or B-cell epitopes, whereas the unique deletion found in the group of subjects without anti-HBs antibodies did not.

**Table 2:** Deletions in large and middle S proteins and association with anti-HBs status.

	Deleted amino acids positions (length)	
Sample	pre-S1 region	pre-S2 region
Concurrent HBsAg/anti-HBs		
LDA 232	64–81 (18 aa)	–
LDA 265	–	22 (1 aa)
LDA 423	–	19–22 (4 aa)
LDA 470	–	17–22 (6 aa)
LDA 489	–	17–22 (6 aa)
HBsAg without anti-HBs		
LDA 405	3–12 (10 aa)	–

Table 3 shows a comparison between deduced amino acids of the large S proteins of the 30 isolates from this study and the consensus sequence of 130 other HBV/E isolates available in the DNA databanks. The 31 amino acid residues shown on Table 3 are those for which a mutation was observed in at least two, but not in all Angolan isolates. Strains derived from four subjects with concurrent HBsAg/anti-HBs (LDA265, LDA423, LDA481 and LDA494) showed an R34K substitution in the pre-S1 region. Furthermore, pre-S2 R16K and/or R18K mutations were observed in five HBV isolates derived from individuals with concurrent HBsAg/anti-HBs (LDA067, LDA232, LDA423, LDA470 and LDA494). At all, mutations disrupting T- or B-cell epitopes, including deletions in the pre-S2 region, substitution R34K in the pre-S1 region, and R to K substitutions at positions 16 and 18 in the pre-S2 region, were observed in 8/12 (67%) samples with concurrent HBsAg and anti-HBs but in none of the 18 samples without anti-HBs antibodies. Otherwise, the Q129H escape mutation in determinant ‘*a*’ was observed in two isolates (LDA265 and LDA339; Table 3).

**Table 3:** Comparison of deduced amino acid sequences (specific positions) of Angolan HBV isolates and HBV/E consensus.

	Consensus amino acids and positions																														
	Pre-S1 region								Pre-S2 region								S region														
Samples	34	38	56	66	76	84	85	90	5	11	16	17	18	19	22	39	3	10	13	14	20	21	45	83	129	164	189	193	195	203	204
	R	R	K	F	W	L	K	D	S	A	R	V	R	G	F	A	S	G	L	V	F	L	A	F	Q	E	T	S	I	P	S
Concurrent HBsAg and anti-HBs																															
LDA067	.	.	.	.	.	.	.	N	.	.	.	.	K	.	.	.	G	.	.	.	.	.	.	.	.	.	.	.	.	.	.
LDA072	.	.	.	.	.	.	.	.	.	.	.	.	.	.	.	V	.	.	.	.	.	.	.	.	.	.	I	.	.	.	.
LDA111	.	.	.	.	.	.	.	.	.	.	.	.	.	.	.	.	.	.	.	.	.	.	.	.	.	.	.	.	.	.	.
LDA232	.	.	.	–	–	.	T	.	A	.	.	.	K	.	.	.	.	.	P	.	S	W	.	.	.	.	.	.	.	.	.
LDA265	K	.	E	.	.	.	T	.	.	.	.	.	.	.	–	.	.	.	.	.	.	.	.	.	H	.	.	.	.	.	.
LDA277	.	.	.	.	.	.	.	.	.	.	.	.	.	.	.	.	N	.	.	.	.	.	.	.	.	.	.	.	.	.	.
LDA278	.	.	.	.	.	.	.	.	.	.	.	.	.	.	.	.	.	.	.	.	.	.	.	.	.	.	.	.	.	.	.
LDA423	K	.	.	.	.	.	T	.	.	.	K	A	K	–	–	.	.	.	.	.	.	.	.	.	.	.	.	.	.	.	.
LDA470	.	.	.	.	S	.	.	N	.	.	K	–	–	–	–	.	.	E	R	.	.	S	.	.	.	.	.	.	.	.	.
LDA481	K	K	.	.	.	.	.	.	.	.	.	.	.	.	.	.	.	.	.	.	.	.	.	.	.	.	I	.	.	Q	N
LDA489	.	.		.	.	.	.	.	.	.	.	–	–	–	–	.	.	.	.	.	.	.	.	.	.	.	.	L	.	.	K
LDA494	K	.	.	I	.	.	.	.	.	.	K	.	.	.	.	.	.	.	.	A	.	S	.	.	.	.	.	.	.	R	.
HBsAg without anti-HBs																															
LDA154	.	.	.	.	.	.	.	.	.	.	.	.	.	.	.	.	.	.	.	.	.	.	.	.	.	.	.	.	.	.	.
LDA173	.	.	.	V	.	.	.	.	.	.	.	.	.	.	.	V	.	.	.	.	.	.	.	.	.	G	.	.	M	.	.
LDA264	.	K	.	V	.	.	.	.	Y	T	.	.	.	.	S	.	.	K	.	.	.	.	.	.	.	.	I	.	.	.	.
LDA274	.	.	.	.	.	.	.	.	.	.	.	.	.	.	.	.	G	.	.	.	.	.	.	.	.	.	.	.	.	.	.
LDA332	.	.	.	.	.	.	.	N	.	.	.	.	.	D	L	.	.	.	.	.	.	.	.	.	.	G	.	.	.	.	.
LDA335	.	.	.	.	.	.	.	.	.	.	.	.	.	.	.	.	.	.	.	.	.	.	.	.	.	.	.	.	.	.	.
LDA339	.	.	.	.	.	.	T	.	.	.	.	.	.	.	L	.	.	.	.	.	.	.	.	.	H	D	.	L	M	.	.
LDA379	.	.	.	.	.	.	.	.	.	.	.	.	.	.	.	.	.	.	.	.	.	.	S	.	.	.	.	.	.	.	.
LDA349	.	.	.	.	*	.	.	.	.	.	.	.	.	.	.	.	.	.	.	.	.	.	.	.	.	.	.	.	.	.	.
LDA386	.	.	.	.	.	.	.	.	.	.	.	.	.	.	.	.	.	.	.	.	.	.	.	.	.	.	.	.	.	.	.
LDA399	.	K	.	.	.	.	.	.	.	.	.	.	.	.	.	.	N	.	.	.	.	.	.	.	.	.	.	.	.	.	.
LDA405	.	.	.	.	.	Q	.	.	.	.	.	.	.	.	.	.	.	.	.	.	.	.	V	.	.	.	.	.	.	.	.
LDA408	.	K	Q	.	.	.	.	N	.	.	.	.	.	.	.	.	.	.	.	A	S	.	.	C	.	G	I	.	.	.	.
LDA417	.	.	.	.	.	.	.	.	.	.	.	.	.	.	L	.	.	.	.	.	.	.	.	.	.	.	.	.	.	.	.
LDA439	.	.	.	.	.	.	.	.	.	.	.	.	.	.	.	.	G	.	.	.	.	.	.	.	.	.	.	.	.	.	.
LDA445	.	.	.	.	.	Q	.	.	.	.	.	.	.	.	.	.	G	.	.	.	.	.	.	.	.	.	.	.	.	.	.
LDA452	.	.	.	.	.	.	.	.	.	.	.	.	.	.	.	.	N	.	.	.	.	.	.	C	.	.	.	.	.	.	.
LDA504	.	.	.	.	.	.	.	.	.	T	.	.	.	.	.	.	.	.	.	.	.	.	.	.	.	.	.	.	.	.	.

Consensus sequence shown at the top of the table was deduced from the HBV/E full-length sequences available in GenBank. Only positions for which at least two, but not all, Angolan isolates showed differences with the consensus sequence, are shown.

–: deletion; *: stop codon.

Five individuals were coinfected with HIV-1. At the time of blood collect, three of them (LDA265, LDA173 and LDA339) were under treatment with highly active antiretroviral therapy (HAART) while the two others did not know they were infected with HIV. All three HBV isolates derived from patients under HAART displayed drug resistance mutations. HBV isolate LDA265 showed the lamivudine resistance substitution rtL180M associated to the adefovir resistance mutation rtA181V (not shown). The two others, LDA173 and LDA339, displayed the lamivudine resistance triple mutation rtV173L, rtL180M, rtM204V/I which causes the concomitant amino acid substitutions E164D/G and I195M in the small S protein (Table 3).

The complete genomes of 16/30 HBV isolates (essentially those whose viral load was >10^4^ copies/ml) were successfully amplified by PCR, and their entire nucleotide sequences were determined. Table 4 shows the HBeAg/anti-HBe status, viral load and core promoter/precore mutations of the 16 isolates. The common variations A1762T-G1764A in the basal core promoter and G1896A and G1899A in the precore region were identified in three, four and two isolates, respectively. Among the samples without detectable anti-HBs antibodies, a perfect correlation occurred between precore/core promoter mutations and anti-HBe phenotype, since the three mutated strains (LDA274, LDA399 and LDA417) were precisely those infecting the HBeAg negative, anti-HBe positive subjects. However, such a correlation was not observed among the samples with concurrent HBsAg and anti-HBs, since only the isolate showing both precore and core promoter mutations (LDA423) was derived from an anti-HBe positive individual (Table 4). In the case of sample LDA072 showing a stop codon at nucleotide 1896, a possible explanation for HBeAg detection is that a minority subpopulation of wild-type viruses, undetectable by direct nucleotide sequencing, continues to produce HBeAg.

**Table 4:** HBeAg/anti-HBe status, viral load and precore/core mutations of Angolan subjects (completely sequenced HBV isolates).

			Mutations	
Samples	HBeAg/antiHBe status	Viral load (log copies/ml)	Basal core promoter	Precore
*Concurrent HBsAg and anti-HBs*				
LDA072	Pos/Neg	6.37	–	G1896A
LDA265	Pos/Neg	6.24	A1762T/G1764A	–
LDA277	Pos/Neg	8.25	–	–
LDA278	Neg/Neg	5.33	–	–
LDA423	Neg/Pos	4.46	A1762T/G1764A	G1896A, G1899A
*HBsAg without anti-HBs*				
LDA154	Pos/Neg	9.50	–	–
LDA173	Pos/Neg	7.45	–	–
LDA274	Neg/Pos	4.49	–	G1896A, G1899A
LDA379	Pos/Neg	8.71	–	–
LDA386	Pos/Neg	8.90	–	–
LDA399	Neg/Pos	4.52	A1762/G1764A	–
LDA417	Neg/Pos	5.04	–	G1896A
LDA439	Pos/Neg	7.40	–	–
LDA445	Pos/Neg	5.11	–	–
LDA405	Neg/Neg	5.01	–	–
LDA504	Pos/Neg	4.30	–	–

### Specific Mutations and Phylogenetic Analysis

Alignment of the amino acid sequences of surface antigen, polymerase, core and X proteins of 213 HBV/E isolates from 12 African countries, including those from this study, revealed the existence of five specific mutations, namely Ile57 in the small S protein, His177, Trp245 and Leu612 in the DNA polymerase, and Leu30 in the X protein. These mutations appeared at high frequencies (79–96%) among the isolates from Angola, Namibia and DRC, but at low rates (0–12%) in those from nine other African countries (Cameroon, Central African Republic, Côte d'Ivoire, Ghana, Guinea, Madagascar, Niger, Nigeria and Sudan; Table 5). In all cases, those differences of frequencies were extremely statistically significant (*P*<0.0001).

**Table 5:** Frequencies of atypical amino acids in HBV/E isolates from different African countries.

		Frequency in isolates from		
Amino acid/Position	Protein	Angola, Namibia and DRC^a^	Other African countries^b^	*P* value
Ile57	Small S	27/28 (96%)	12/185 (6%)	<0.0001
His177	Polymerase	22/28 (79%)	0/185 (0%)	<0.0001
Trp245	Polymerase	22/28 (79%)	13/185 (7%)	<0.0001
Leu612	Polymerase	24/28 (86%)	22/185 (12%)	<0.0001
Leu30	X	24/28 (86%)	1/185 (1%)	<0.0001

a Democratic Republic of Congo.

b Cameroon, Central African Republic, Côte d'Ivoire, Ghana, Guinea, Madagascar, Niger, Nigeria and Sudan.

**Figure 1:**
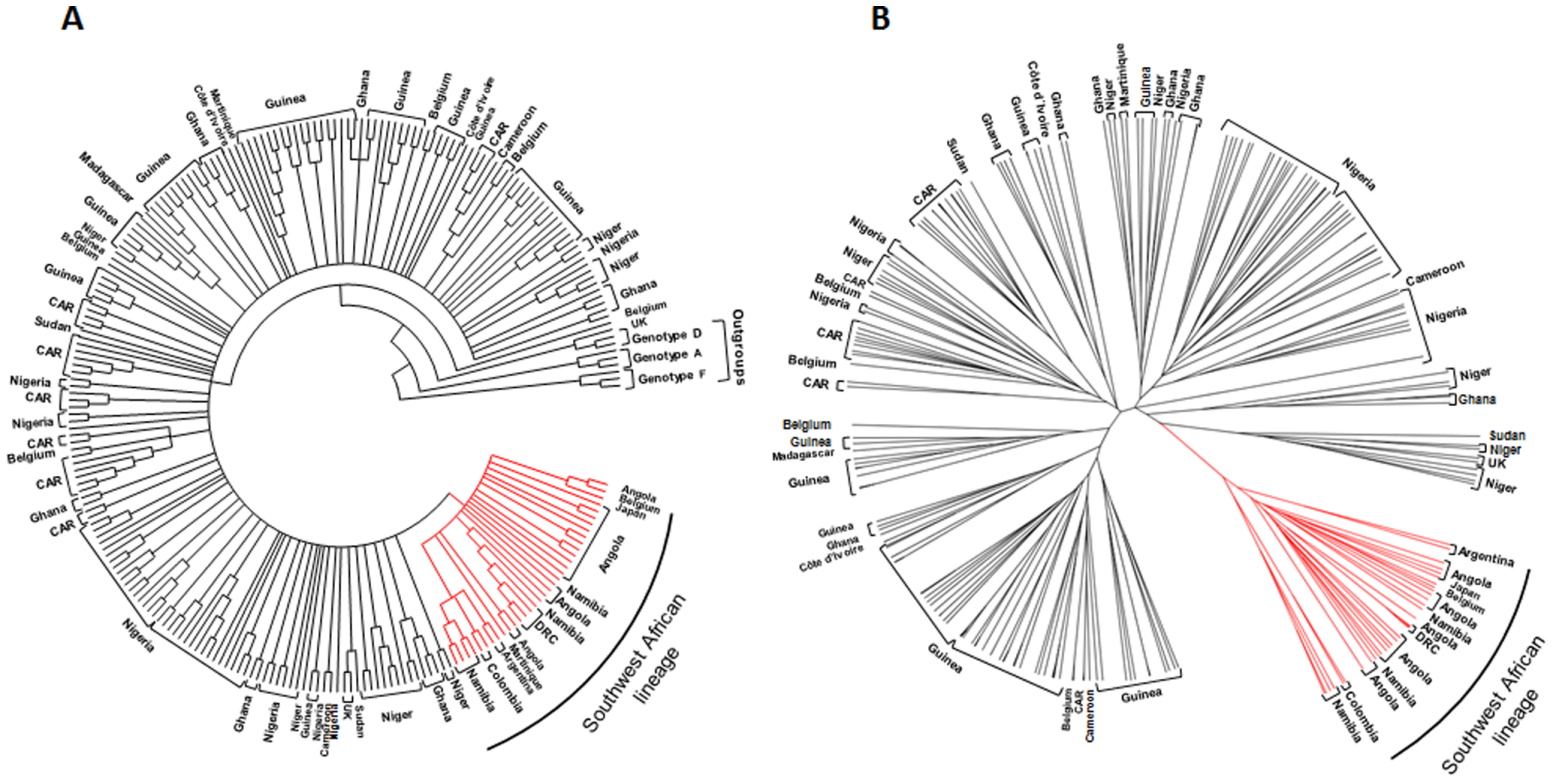
Evidence for a distinct lineage among HBV/E isolates. Phylogenetic trees incorporate 231 HBV/E isolates whose complete nucleotide sequences were available in GenBank, including the 16 complete sequences of Angolan samples described in this study. The list of the isolates (GenBank accession numbers) is available in Table S1. Phylogenetic analyses were performed by (A) the maximum likelihood method and (B) by Bayesian Inference using the Bayesian Markov chain Monte Carlo (MCMC) statistical framework. Southwest African lineage shown in red ink was established with a posterior probability = 1.

**Figure 2:**
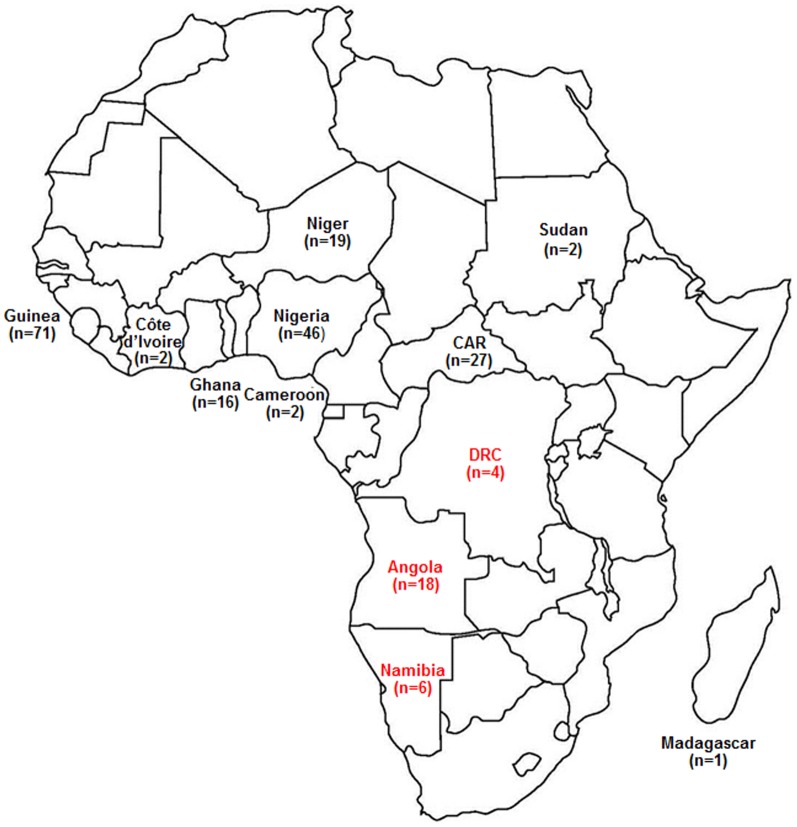
Distribution by country of the African HBV/E isolates completely sequenced. Numbers in parentheses represent the number of full-length sequenced strains. Isolates that belong to the Southwest African lineage are in red while the others are in black. The two types of isolates were not found circulating together in any country. CAR  =  Central African Republic. DRC  =  Democratic Republic of Congo.

Phylogenetic analysis was performed by using the maximum likelihood method with 231 complete HBV/E sequences available in GenBank (including the sequences from this work), a large majority of them from African isolates. Phylogenetic tree (Fig. 1A) showed that all the 28 isolates from Angola, Namibia and DRC clustered separately from all other HBV/E samples isolated in Africa. This cluster was thus called “Southwest African lineage”. Of 15 non African HBV/E isolates, seven (two Colombian, two Argentinian, one Japanese, one Belgian, and one Martinican) clustered in this group, but eight (five Belgian, two British and one Martinican) did not. Bayesian analysis was carried out to confirm the reliability of the Southwest African lineage. Fig. 1B shows that similar results were achieved. The existence of the Southwest African lineage was confirmed with a posterior probability = 1. The intralineage sequence divergence was 0.96%, while the divergence between the isolates belonging to the separate lineage and the others was 1.53%. Fig. 2 shows the localization of the 12 African countries for which at least one complete HBV/E sequence has been deposited in GenBank. Two well distinct geographic areas were observed. No isolate belonging to the separate lineage (represented in red ink) circulates in an African country other than Angola, Namibia or DRC. Inversely, no isolate clustering out of the Southwest African lineage (black ink) circulates in any of the three countries.

## Discussion

In this study, 30 Angolan HBV/E isolates were genetically characterized. Deduced amino acid sequences showed the presence of a methionine residue at position 83 in the C-terminal end of the pre-S1 region of 29/30 (97%) isolates. As the number of sequenced HBV/E samples grows, it is becoming clear that Met83 constitutes a molecular signature of genotype E [23]. It would therefore be important to determine, in future studies, if the corresponding additional AUG codon acts as a start codon, leading to the translation of an elongated middle hepatitis B surface protein (MHBs) and/or if its translation may interfere with the translation of the regular MHBs coded for downstream [23].

Some previous studies have addressed the simultaneous presence of serum HBsAg and anti-HBs in chronic hepatitis B patients, with rates varying from 3–6% in South Korea [6], [24] to 21% in Singapore [5], 24–32% in the United States [3], [4] and 36% in The Netherlands [2]. In this study, the individuals showing concurrent HBsAg and anti-HBs represented 40% of all HBV carriers, with a 95% confidence interval of 30–51%. The reason why such a high proportion was observed in the Angolan population remains unclear. Deletions varying in length from 1 to 18 aminoacids were shown in the pre-S region of six isolates (Table 2). Deletions located between aa residues 19 and 22 in the pre-S2 region, detected in four strains, had already been described in HBV/E isolates from Benin, Cameroon, DRC, Mali, Nigeria and Togo [19]. Interestingly, all five deletions detected in samples with concurrent HBsAg and anti-HBs were found to disrupt viral epitopes recognized by T- or B-cells, whereas the sixth one, observed in an isolate infecting an anti-HBs negative individual, did not. The simultaneous detection of surface antigens and antibodies may be correlated to the disruption of epitopes in large S and middle S proteins, which could diminish or even completely abolish the recognition of the modified HBsAg by the antibodies of the infected subjects [21], [22]. Here, substitutions (notably R34K in the pre-S1- and R16K and R18K in the pre-S2 region) were noted which were only present in samples with concurrent HBsAg and anti-HBs and may be responsible for this phenotype. Further studies are warranted to determine whether the high proportion (40%) of individuals with concurrent HBsAg/anti-HBs found among Angolan subjects is related to their clinical status (asymptomatic carriers), to the fact that Angola is an area of hyperendemicity for HBV infection, or to any other reason.

Although described for the first time two decades ago [12], HBV genotype E has not been studied as extensively as other genotypes, due to its spreading be mainly restricted to Africa. While genotype A dominates in Eastern and South-Eastern Africa, HBV/E is the predominant genotype in the West African crescent that extends from Senegal to Namibia [20], [25], [26], as well as in CAR [27], DRC [19] and Madagascar [28]. Out of Africa, HBV/E isolates have been identified in Brazil [29], [30], Argentina [31], Colombia [18], the Caribbean island of Martinique [32], Haiti [33], France [34], [35], and Belgium [36]. All cases were related to the migration of African people to other continents. Additionally, three HBV/E isolates derived from native Belgian persons have also been described [37]. The fact that only few HBV/E isolates have been identified in the Americas is compatible with a relatively recent introduction into humans, after the forced slave migration during the centuries 16th to 19th [19], [26], or with the hypothesis that HBV/E remained confined to isolated African communities for a long period of time and reintroduced into the general African population in the last 200 years [38]. This would also explain the relatively low genetic diversity of HBV/E isolates (mean divergence of 1.75% over the whole genome) [19], [23]. This low variability has not allowed the subdivision of genotype E into subgenotypes, as was done with genotypes A–D and F, since such a subdivision would require a mean divergence of about 4% between subgenotypes, as proposed previously [15], [39].

By comparing the different countries of the West-African crescent, a previous study has pointed out a genetic diversity among the HBV/E isolates lower in the Southern countries than in the Northern ones [40]. In the present study, performed with Angolan samples, the presence of characteristic amino acid residues, namely Ile57 in the small S protein, His177, Trp245 and Leu612 in the polymerase, and Leu30 in the X protein, was noted in the large majority of the strains. More generally, the frequency of these residues was much higher among the HBV/E isolates from Angola, Namibia and DRC (79–96%) than in those from Cameroon, CAR, Côte d'Ivoire, Ghana, Guinea, Madagascar, Niger, Nigeria and Sudan (0–12%; Table 5). Furthermore, phylogenetic analysis performed with two independent methods resulted in trees showing the existence of a distinct lineage, called Southwest African lineage, with a Bayesian posterior probability = 1 (Fig. 1). Intragroup divergence value was 0.96% within Southwest African lineage, while the mean genetic distance between the isolates belonging to this specific lineage and the others was 1.53%. Within Africa, geographical distribution of that lineage was restricted to Angola, Namibia and DRC while the HBV/E isolates not belonging to that lineage were disseminated in eight countries of the equatorial region and Madagascar. The co-circulation of HBV/E isolates belonging and not belonging to the separate lineage was not observed in any African country (Fig. 2). The non-African isolates were found both inside and outside the group. This was not surprising if we consider that all these samples have been described as originating from African travelers, emigrants or African descendants of the first generation.

In conclusion, the results reported here confirm previous observations showing a lower genetic diversity of the HBV/E isolates circulating in Angola, Namibia and DRC when compared to those of other African countries, and demonstrate the existence of a separate HBV/E lineage circulating in those three, but not in other nine, African countries.

## Materials and Methods

### Ethics Statement

Participation was voluntary, written informed consent was obtained, and participants were offered post-test counseling. Ethical approval was obtained from the Ethics Committees of the Ministry of Health, Angola (November 2007) and the Oswaldo Cruz Foundation, Brazil (project 362/07).

### HBV/E Samples

Seventy-seven (15.2%) out of 508 blood samples, collected in 2007 from staff and visitors of a private hospital (Divina Providência) in Luanda, Angola, were HBsAg positive. Among these, 31 (40.3%) had concurrent HBsAg and anti-HBs antibodies. HBV DNAs of 41/77 HBsAg positive samples were successfully amplified by PCR. Of these, 13 (32%) were both HBsAg and anti-HBs positive. By PCR-RFLP, it was shown that 35/41 virus isolates belonged to genotype E (HBV/E) [20].

### PCR Amplification and Calculation of the Viral Load

HBV DNA was extracted from 0.2 ml of serum sample using *High Pure Viral Nucleic Acid kit* (Roche Diagnostics, Mannheim, Germany) according to the manufacturer's instructions. Quantification of HBV DNA was performed by using TaqMan real-time PCR technology, as described previously [41]. The detection limit of the assay was 10 copies/reaction, corresponding to about 100 copies/ml of serum. The whole genomes of 16 HBV/E isolates were successfully amplified by using a previously described method [42]. The 19 remaining HBV/E samples had their pre-S/S region amplified by nested PCR as described in a previous report [20].

### Nucleotide Sequencing

Nucleotide sequences of full-length HBV genomes were determined by direct sequencing using the *BigDye Terminator v3.1 Cycle Sequencing kit* (Applied Biosystems, Foster City, CA) and a set of specific HBV primers [43]. Sequencing reactions were analyzed on an ABI 3730 automated sequencer (Applied Biosystems). The nucleotide sequences have been deposited in the GenBank database (accession numbers HM195104, HM195106, HM195107, HM195109, HM195112, and KF849713 to KF849737.

### Phylogenetic analysis

Multiple sequence alignment was performed by using Clustal X program [44] with 231 complete HBV/E sequences available in GenBank (see list and origin of the sequences in Table S1). Phylogenetic analysis was carried out (i) using the maximum likelihood method (bootstrap resampling test with 1,000 replicates) in MEGA version 5.1 software and (ii) by Bayesian Inference using the Bayesian Markov chain Monte Carlo (MCMC) statistical framework implemented in the BEAST v1.7.4 package [45] under the model of nucleotide substitution GTR+Γ+I which was selected as the best-fit model by the jModeltest program [46]. MCMC analysis was run for 1×10^8^ generations to achieve the convergence of parameters, which was assessed after 25% burn-in and calculation of Effective Sample Size (ESS) using TRACER v1.5 [47]. All parameters estimates showed ESS values >200 and their uncertainties were reflected in the 95% Highest Posterior Density intervals. The maximum clade credibility was visualized with FigTree v1.3.1 program [48] after the posterior tree distribution had been summarized using the TreeAnnotator v.1.7.4 program.

## Supporting Information

Table S1 **Nucleotide sequences used in this work.** List of the HBV genotype E complete nucleotide sequences (GenBank accession numbers), classified by country, used to construct the phylogenetic trees. The following criteria were used to include the sequences in the phylogenetic studies: non recombinant human isolates from known country whose nucleotide sequences have been totally determined and did not show any insertion.
(DOCX)
