# Supplementary material for: Analysis of Complete Nucleotide Sequences of Angolan Hepatitis B Virus Isolates Reveals the Existence of a Separate Lineage within Genotype E
Source: PLoS One. 2014 Mar 14;9(3):e92223. doi: 10.1371/journal.pone.0092223 (PMC3954871; doi:10.1371/journal.pone.0092223)
Supplement: Table S1 — Nucleotide sequences used in this work. List of the HBV genotype E complete nucleotide sequences (GenBank accession numbers), classified by country, used to construct the phylogenetic trees. The following criteria were used to include the sequences in the phylogenetic studies: non recombinant human isolates from known country whose nucleotide sequences have been totally determined and did not show any insertion. (DOCX) [file pone.0092223.s001.docx]

Table S1. Nucleotide sequences used in this work

|  | | | | | | | | | | | | |
| --- | --- | --- | --- | --- | --- | --- | --- | --- | --- | --- | --- | --- |
| **Angola, 18 isolates** | | | | | | | | | | | | |
| DQ060822 | | | DQ060823 | | | KF849713 | | KF849714 | | | KF849715 | |
| KF849716 | | | KF849717 | | | KF849718 | | KF849719 | | | KF849720 | |
| KF849721 | | | KF849722 | | | KF849723 | | KF849724 | | | KF849725 | |
| KF849726 | | | KF849727 | | | KF849728 | |  | | |  | |
| **Argentina, 2 isolates** | | | | | | | | | | | | |
| JQ000008 | | | JQ000009 | | |  | |  | | |  | |
| **Belgium, 7 isolates** | | | | | | | | | | | | |
| AY935700 | | | FJ349226 | | | FJ349227 | | FJ349237 | | | FJ349238 | |
| FJ349239 | | | FJ349240 | | |  | |  | | |  | |
| **Cameroon, 2 isolates** | | | | | | | | | | | | |
| AB194947 | | | AB194948 | | |  | |  | | |  | |
| **Central African Republic (CAR), 27 isolates** | | | | | | | | | | | | |
| AM494689 | | | AM494690 | | | AM494691 | | AM494692 | | | AM494693 | |
| AM494694 | | | AM494696 | | | AM494697 | | AM494698 | | | AM494699 | |
| AM494700 | | | AM494701 | | | AM494702 | | AM494703 | | | AM494704 | |
| AM494705 | | | AM494706 | | | AM494707 | | AM494708 | | | AM494709 | |
| AM494710 | | | AM494711 | | | AM494712 | | AM494713 | | | AM494714 | |
| AM494715 | | | AM494717 | | |  | |  | | |  | |
| **Colombia, 2 isolates** | | | | | | | | | | | | |
| JQ023664 | | | JQ023665 | | |  | |  | | |  | |
| **Côte d’Ivoire, 2 isolates** | | | | | | | | | | | | |
| AB091255 | | | AB091256 | | |  | |  | | |  | |
| **Democratic Republic of Congo, 4 isolates** | | | | | | | | | | | | |
| AY738144 | | | AY738145 | | | AY738146 | | AY738147 | | |  | |
| **Ghana, 16 isolates** | | | | | | | | | | | | |
| AB205129 | | | AB205188 | | | AB205189 | | AB205190 | | | AB205191 | |
| AB205192 | | | AB106564 | | | EU239217 | | EU239219 | | | EU239220 | |
| EU239221 | | | EU239222 | | | EU239223 | | EU239224 | | | EU239225 | |
| EU239226 | | |  | | |  | |  | | |  | |
| **Guinea, 71 isolates** | | | | | | | | | | | | |
| GQ161755 | | | GQ161757 | | | GQ161758 | | GQ161759 | | | GQ161760 | |
| GQ161761 | | | GQ161762 | | | GQ161763 | | GQ161764 | | | GQ161765 | |
| GQ1617666 | | | GQ161768 | | | GQ161769 | | GQ161770 | | | GQ161771 | |
| GQ161772 | | | GQ161773 | | | GQ161774 | | GQ161776 | | | GQ161777 | |
| GQ161778 | | | GQ161779 | | | GQ161780 | | GQ161781 | | | GQ161782 | |
| GQ161783 | | | GQ161784 | | | GQ161785 | | GQ161786 | | | GQ161787 | |
| GQ161789 | | | GQ161790 | | | GQ161791 | | GQ161792 | | | GQ161793 | |
| GQ161794 | | | GQ161795 | | | GQ161796 | | GQ161797 | | | GQ161798 | |
| GQ161799 | | | GQ161800 | | | GQ161801 | | GQ161802 | | | GQ161803 | |
| GQ161804 | | | GQ161807 | | | GQ161808 | | GQ161809 | | | GQ161810 | |
| GQ161811 | | | GQ161812 | | | GQ161814 | | GQ161815 | | | GQ161816 | |
| GQ161817 | | | GQ161819 | | | GQ161820 | | GQ161823 | | | GQ161824 | |
| GQ161825 | | | GQ161826 | | | GQ161827 | | GQ161828 | | | GQ161829 | |
| GQ161831 | | | GQ161832 | | | GQ161833 | | GQ161834 | | | GQ161835 | |
| GQ161836 | | |  | | |  | |  | | |  | |
| **Japan, 1 isolate** | | | | | | | | | | | | |
| AP007262 |  | | | | |  | | |  | | |  |
| **Madagascar, 1 isolate** | | | | | | | | | | | | |
| DQ060830 | | |  | | |  | |  | | |  | |
| **Martinique, 2 isolates** | | | | | | | | | | | | |
| HE974380 | HE974384 | | | | |  | | |  | | |  |
| **Namibia, 6 isolates** | | | | | | | | | | | | |
| DQ060824 | | | | DQ060825 | | DQ060826 | | DQ060827 | | | DQ060828 | |
| DQ060829 | | | |  | |  | |  | | |  | |
| **Niger, 19 isolates** | | | | | | | | | | | | |
| FN594748 | | | FN594749 | | | FN594750 | | FN594751 | | | FN594752 | |
| FN594753 | | | FN594754 | | | FN594755 | | FN594756 | | | FN594757 | |
| FN594758 | | | FN594759 | | | FN594760 | | FN594761 | | | FN594762 | |
| FN594763 | | | FN594764 | | | FN594765 | | FN594766 | | |  | |
| **Nigeria, 46 isolates** | | | | | | | | | | | | |
| HM363565 | HM363566 | | | | | HM363567 | | HM363568 | | | HM363569 | |
| HM363570 | HM363571 | | | | | HM363572 | | HM363573 | | | HM363574 | |
| HM363575 | HM363576 | | | | | HM363577 | | HM363578 | | | HM363579 | |
| HM363580 | HM363581 | | | | | HM363582 | | HM363583 | | | HM363584 | |
| HM363585 | HM363586 | | | | | HM363587 | | HM363588 | | | HM363589 | |
| HM363590 | HM363591 | | | | | HM363592 | | HM363593 | | | HM363594 | |
| HM363595 | HM363596 | | | | | HM363597 | | HM363598 | | | HM363599 | |
| HM363600 | HM363601 | | | | | HM363602 | | HM363603 | | | HM363604 | |
| HM363605 | HM363606 | | | | | HM363607 | | HM363608 | | | HM363610 | |
| HM363611 | | | | | | | | | | | | |
| **Sudan, 2 isolates** | | | | | | | | | | | | |
| KF170741 | | | KF170742 | | |  | |  | | |  | |
| **UK, 3 isolates** | | | | | | | | | | | | |
| AB219529 | | AB219533 | | | AB219534 | |  | | |  | | |
|  | |  | | |  | |  | | |  | | |
